# RNA interference-based strategies to control *Botrytis cinerea* infection in cultivated strawberry

**DOI:** 10.1007/s00299-024-03288-7

**Published:** 2024-07-24

**Authors:** Luca Capriotti, Barbara Molesini, Tiziana Pandolfini, Hailing Jin, Elena Baraldi, Michela Cecchin, Bruno Mezzetti, Silvia Sabbadini

**Affiliations:** 1 Department of Agricultural, Food and Environmental Sciences, Università Politecnica delle Marche, Ancona, Italy; 2 Department of Biotechnology, University of Verona, Strada Le Grazie, 15, 37134 Verona, Italy; 3 Department of Microbiology and Plant Pathology, Center for Plant Cell Biology, Institute for Integrative Genome Biology, University of California, Riverside, CA USA; 4 Department of Agricultural and Food Science, DISTAL, Alma Mater Studiorum - University of Bologna, Bologna, Italy

**Keywords:** *Fragaria* x *ananassa* (strawberry), Gene silencing, Gray mold, *In planta* expression, RNAi, Spray

## Abstract

**Key message:**

Gene silencing of *BcDCL* genes improves gray mold disease control in the cultivated strawberry.

**Abstract:**

Gene silencing technology offers new opportunities to develop new formulations or new pathogen-resistant plants for reducing impacts of agricultural systems. Recent studies offered the proof of concept that the symptoms of gray mold can be reduced by downregulating *Dicer-like 1* (*DCL1*) and *2* (*DCL2*) genes of *Botrytis cinerea*. In this study, we demonstrate that both solutions based on dsRNA topical treatment and *in planta* expression targeting *BcDCL1* and *BcDCL2* genes can be used to control the strawberry gray mold, the most harmful disease for different fruit crops. 50, 70 and 100 ng μL^−1^ of naked *BcDCL1/2* dsRNA, sprayed on plants of *Fragaria* x *ananassa* cultivar Romina in the greenhouse, displayed significant reduction of susceptibility, compared to the negative controls, but to a lesser extent than the chemical fungicide. Three independent lines of Romina cultivar were confirmed for their stable expression of the hairpin gene construct that targets the *Bc-DCL1* and* 2* sequences (hp-Bc-DCL1/2), and for the production of hp construct-derived siRNAs, by qRT-PCR and Northern blot analyses. In vitro and in vivo detached leaves, and fruits from the hp-Bc-DCL1/2 lines showed significantly enhanced tolerance to this fungal pathogen compared to the control. This decreased susceptibility was correlated to the reduced fungal biomass and the downregulation of the *Bc-DCL1* and *2* genes in *B. cinerea*. These results confirm the potential of both RNAi-based products and plants for protecting the cultivated strawberry from *B. cinerea* infection, reducing the impact of chemical pesticides on the environment and the health of consumers.

**Supplementary Information:**

The online version contains supplementary material available at 10.1007/s00299-024-03288-7.

## Introduction

One of the greatest challenges for farmers is to ensure sufficient and safe food in an economically, socially and environmentally sustainable way. Plant diseases are one of the main problems affecting several crop species, which are mainly controlled by the application of pesticides (Sharma et al. 2019). In fact, there is strong social and political pressure to decrease the use of agrochemicals, as also highlighted in the recently published European Green Deal, where a 50% decrease in the use of toxic pesticides is expected before 2030 (Tataridas et al. 2022). To achieve this goal, it is essential to find new alternative strategies to protect crops from endemic pests and other phytosanitary emergencies such as those due to climate change (Rosa et al. 2022).

Strawberry (*Fragaria* x *ananassa*) industry is continuously increasing its importance in the sector of worldwide horticultural production, estimated at 14 billion USD in 2020 (Hernández-Martínez et al. 2023). This fruit is particularly appreciated for its taste and nutraceutical properties, being rich in vitamins, anthocyanins, and polyphenols (Mazzoni et al. 2020). Unfortunately, for the consumer, the strawberry is also perceived as a fruit with risk of possible high contamination of pesticides, as it is very susceptible to various pathogens and parasites. Among the pathogens that affect this soft fruit, *Botrytis cinerea*, the causal agent of gray mold disease, is one of the most impactful from an economic point of view, by challenging strawberry at flowering/fruiting and post-harvest stages, with losses estimated at around 80% under proper fungal growth conditions, in plants not treated with fungicides (Petrasch et al. 2019).

As for all crops, also for strawberry, one possible strategy is the development of varieties with increased tolerance to diseases obtained through classical breeding techniques. However, resistances obtained through traditional methods show little stability and durability, and this solution is not always feasible due to the lack of resistance genes (McDonald 2014; Willocquet et al. 2017; Senger et al. 2022).

Modern agricultural biotechnology is continuously advancing and improving its tools, aiming to successfully address various challenges (Sabbadini et al. 2021a), including the reduction of agrochemicals. RNA interference (RNAi) has proven to be a powerful and precise biotechnological tool that can be exploited for plant genetic improvement and protection (Mezzetti et al. 2020). RNAi is a well-known natural biological process taking part in the gene regulation process of all eukaryotes, whereby double-stranded RNA (dsRNA) molecules regulate gene expression in a sequence-dependent manner. RNAi is triggered by small non-coding RNAs (sRNAs), generated by the Dicer-like endoribonuclease (DCL) activity on dsRNA molecules. Mature sRNAs are loaded into RNA-induced silencing (RISC) complex leading to the degradation of homologous transcripts (Hung and Slotkin 2021; Zand Karimi and Innes 2022).

The expression of dsRNA-generating constructs in plants, through a genetic engineering approach, permits the silencing of pathogen genes, a phenomenon called host-induced gene silencing (HIGS), conferring a constitutive resistance/tolerance to diseases (Wang et al. 2016; Ghag 2017; Koch and Wassenegger 2021). Recent research has demonstrated that a communication mechanism based on sRNA trafficking via extracellular vesicles (EVs) occurs between fungal pathogens and host plants during infection (Cai et al. 2018; He et al. 2021, 2023; Qiao et al. 2023). Such a mechanism, called bi-directional cross-kingdom RNAi, seems to be crucial to modulate both the host defense response and the fungal pathogenicity at early stages of the interaction, which elucidates the mechanistic basis for HIGS. HIGS approach has been exploited over the last decades to reduce pathogenicity and growth of various plant pathogens, including fungi (Nowara et al. 2010; Koch et al. 2013; Ghag et al. 2014; Cheng et al. 2015; Wang et al. 2016; Song and Thomma 2018; Qi et al. 2018; Guo et al. 2019; Dou et al. 2020; Wang and Dean 2022; Wytinck et al. 2022), viruses (Reyes et al. 2011; Sidorova et al. 2021; Singh et al. 2021; Alburquerque et al. 2023), oomycetes (Sanju et al. 2015; Jahan et al. 2015; Govindarajulu et al. 2015; Cheng et al. 2022), and insects (Thakur et al. 2014; Guo et al. 2014; Abdellatef et al. 2015; Mamta et al. 2016; Yang et al. 2021; Bao et al. 2021; Adeyinka et al. 2023; Zhang et al. 2024).

Recently, many aggressive fungal pathogens were found to be capable of taking up RNAs from the environment (Koch et al. 2016; Wang et al. 2016; Qiao et al. 2021). These discoveries open new possibilities of pathogen control through the exogenous application (e.g., spray) of RNAi-based biopesticides on plants with a targeted specific effect on pathogens, an approach called spray-induced gene silencing (SIGS) (Chen et al. 2023).

Recently, it has been shown that targeting RNAi core proteins of some pathogens can reduce their virulence on specific crops (Wang et al. 2016; Werner et al. 2020; Haile et al. 2021; Qiao et al. 2021). In particular, since *B. cinerea* can deliver small RNAs via EVs into plant cells to silence host immunity genes (Cai et al. 2018; He et al. 2023), the stable expression of dsRNAs that target *Botrytis cinerea Dicer-like 1* (*Bc-DCL1*) and *Bc-DCL2* genes in *Arabidopsis*, resulted in silencing of *Bc-DCL* genes and limited fungal growth. In addition, the application of sRNAs or dsRNAs that target *Bc-DCL 1* and* 2* genes directly on plant tissues, including strawberry detached fruits, significantly inhibited gray mold disease (Wang et al. 2016; McLoughlin et al. 2018; Qiao et al. 2021). Application of *E. coli*-derived anucleated minicells loaded with dsRNA targeting the *DCL1* and *DCL2* genes of *B. cinerea* halted disease progression on strawberries for up to 12 days in greenhouse conditions (Islam et al. 2021).

The present study aims to demonstrate the effectiveness of dsRNAs targeting *Bc-DCL 1* and* 2* genes by exogenous treatment, as bio-fungicide, of strawberry (*Fragaria* x *ananassa*) plants grown in the greenhouse, and by generating *Bc-DCL 1/2* dsRNA stable expressing plants. Besides confirming, by in vitro and in vivo experiments, the ability of *Bc-DCL1/2*-dsRNAs to reduce the development of the disease, our results show that both these RNAi strategies can be considered efficient alternatives for increasing the sustainability and safety of fresh strawberry production.

## Materials and methods

### Exogenous application of dsRNAs on wild-type strawberry plants cultivated in the greenhouse

“Tray” plants of *Fragaria* x *ananassa* cv Romina, previously conserved at −1 °C for 6 months to induce flower differentiation, were cultivated in 14 cm diameter pots containing commercial peat, and grown in the greenhouse with a photoperiod of 16 h light and at controlled temperature of 25 ± 2 °C. When the plants started to produce ripe fruits, a set of dsRNA-based solutions were prepared following the protocol described in Sabbadini et al., (2021b), to carry out topical applications on strawberry plants. In particular, three different solutions of naked dsRNA molecules synthesized by AgroRNA (Genolution, Seoul, Korea), corresponding to the 490 bp *Bc-DCL 1/2* sequence by Wang et al. (2016), were diluted with deionized sterile water to reach three different concentrations (50, 75 and 100 ng μL^−1^). Similarly, long dsRNA molecules targeting 467 bp of the *eGFP* sequence, from base 157 to base 624, were provided by the same external service, and three solutions at 50, 75, and 100 ng μL^−1^ concentrations were prepared and used as negative controls. All the dsRNA-based solutions were also supplemented with 0.025% Silwet. The commercial fungicide SWITCH (Syngenta) at a concentration of 0.8 g L^−1^ (manufacturer instruction) and deionized sterile water were used as additional positive and negative controls, respectively.

A total of five Romina plants were sprayed, using a low-pressure sprayer, with each of the solutions described above. All leaves and fruits of each plant were sprayed paying attention to cover their whole surface (about 10 mL solution per plant), avoiding cross-contaminations among treatments through the use of cardboard panels placed among plants rows.

One day after treatment, plants were sprayed with a solution composed of 0.1 g L^−1^ of potato dextrose broth (PDB), and 10^5^ conidia mL^−1^ of *B. cinerea* B05.10 strain, collected from 10 days fungus culture growing on MEA (Malt Extract Agar) Petri plates, adding 15 mL of distilled-sterile water up each Petri dish, scratching the surface with sterile inoculation loop (10 μL^−1^) and filtering the solution using a cell strainer 70 μm Nylon mesh (Fischerbrand), specific for conidia selection.

A complete randomized experimental design was used with three replications and 15 plants per treatment. Data on disease severity were acquired, through “masking” (blinding) of the investigator (Karp et al. 2022), on sprayed fruits 4, 7, and 14 days after *B. cinerea* infection (Supplementary Fig. S1a, b). The disease severity index was expressed as a percentage of the infected area on the total area of each fruit at each observation, attributing specific class of diseases: 0 = no disease symptoms; 1 = 0.1–5%; 2 = 5.1–20%; 3 = 20.1–40%; 4 = 40.1–100% (Supplementary Fig. S1c). The disease severity percentage was calculated applying the following formula, as described in Kim et al. (2007): Disease severity (%) = ((Σ (the number of diseased fruits × disease severity index))/(4 × the number of fruits rated)) × 100.

At each time observation and for each treatment, the relative disease control value (%) has been obtained by calculating the difference in disease severity between the negative control (deionized water) and the specific treatment, dividing that value by the disease severity of the negative control, all expressed as a percentage.

### *Agrobacterium*-mediated hp-Bc-DCL1/2 plant production and growth conditions

The engineered *Agrobacterium tumefaciens* strain GV3101, harboring the pHellsgate–*Bc*-*DCL1/2* gene construct (Wang et al. 2016), was used to generate strawberry cv Romina (*Fragaria* x *ananassa*) plants expressing the hp-Bc-DCL1/2 construct. Expanded leaves of 1-month-old in vitro strawberry shoots were used as starting explants for the transformation trial, following the protocol described by Cappelletti et al., (2015), and placed after infection in the growth chamber at 16 h of light at an intensity of 70 μmol m^−2^ s^−1^. Shoots were selected in an optimized regeneration medium composed of MS salts and vitamins (Murashige and Skoog 1962), 30 g L^−1^ sucrose, 7 g L^−1^ plant agar, supplemented with 0.5 mg L^−1^ Thidiazuron, 0.02 mg L^−1^ 2,4-Dichlorophenoxyacetic acid, 200 mg L^−1^ cefotaxime, and 10 mg L^−1^ kanamycin (Duchefa Biochemie) as selective agent, at pH 5.8.

Regenerating shoots were isolated and proliferated on MS medium supplemented with 0.25 mg L^−1^ N6-benzylaminopurine and 10 mg L^−1^ kanamycin, then elongated and rooted on the same cytokinin-free medium, together with the wild-type control. Shoots were then acclimatized in December 2020, first in 60-cell paper pots with highly humid environmental conditions, and finally transferred to greenhouse-controlled conditions in 14 cm diameter pots containing commercial peat.

### PCR analysis of hp-Bc-DCL1/2 strawberry regenerated lines

PCR analyses were performed on in vitro rooted lines to amplify 340 bp of the 35S promoter controlling the expression of the hp-Bc-DCL1/2 sequence, using the dilution protocol of the Thermo Scientific Phire Plant Direct PCR Kit (Fisher Scientific) following the procedure described in Capriotti et al. (2023) with few modifications. This sequence was amplified with the following primers: 35S-F, 5’-CTTCGTCAACATGGTGGAGCACGACA-3’ and 35S-R, 5’- TGGAGATATCACATCAATCCACTT-3’, and the PCR conditions were as follows: 98 °C for 5 min; 40 cycles at 98 °C for 5 s, 63.6 °C for 5 s, 72 °C for 20 s, followed by 72 °C for 1 min. The plasmid DNA of pHellsgate–*Bc*-*DCL1/2* was used as the positive control, while the DNA from wild-type Romina in vitro leaf explants was used as the negative control. 10 μL of amplified sequences were loaded on agarose gel (1%, w/v) with SYBER® Safe DNA Gel Stain (Invitrogen) and detected by UV after electrophoresis.

### Expression analysis of hp-Bc-DCL1/2 plants and siRNA accumulation

Total RNA was isolated from leaves of WT and hp-Bc-DCL1/2 lines following the protocol of Gambino et al., (2008). Approximately 12 μg was separated on 0.8% agarose-formaldehyde denaturing gel. Blotting was performed overnight in the presence of SSC10X buffer on Hybond N + membrane (GE Healthcare). The DNA probe, corresponding to the entire arm of the hairpin construct, was labeled with (α-^32^P)-dCTP using the “Random primed DNA labeling kit” (Roche). ProbeQuant™ G-50 microcolumns (GE Healthcare) were used to purify the labeled probe from unincorporated nucleotides. 10^6^ cpm mL^−1^ of labeled probe was added to ULTRAhyb buffer (Ambion). The membrane was hybridized overnight at 42 °C and then washed twice in 2X Saline-Sodium Citrate (SSC)/0.1% Sodium Dodecyl Sulfate (SDS) for 5 min and twice in 0.1X SSC/0.1% SDS for 15 min at 42 °C. Enrichment of the fraction of small RNAs from total RNAs, extracted as previously described, was performed as reported in Pandolfini et al. 2003, with minor modifications. About 40 μg was separated on 15% polyacrylamide-7 M urea gels and transferred to a Hybond N + membrane (GE Healthcare) by electroblotting for 1 h at 100 V in 0.5X Tris Borate EDTA (TBE) buffer. The single-stranded RNA probe corresponding to the first arm of the hp-Bc-DCL1/2 genetic construct was obtained by in vitro transcription with the TranscriptAid T7 High Yield Transcription kit (Thermo Scientific) in the presence of (α-^32^P)-CTP. After treatment with DNAseI, the probe was hydrolyzed with alkaline buffer (80 mM sodium bicarbonate, 120 mM sodium carbonate) at 60 °C for 3 h. The time of hydrolysis was calculated following Hamilton and Baulcombe (1999). The blot was hybridized overnight at 40 °C in ULTRAhyb; then washed two time in 2X SSC and 0.2% SDS at 50 °C. To check the homogeneity of transfer of the loaded RNA samples after blotting, filters were stained with 0.1% toluidine blue. Detection of ^32^P was performed with Carestream Kodak BioMax XAR films (Sigma-Aldrich). For quantitative reverse transcription PCR (qRT-PCR), cDNA was synthesized using 1 μg of total RNA extracted from leaves, oligo (dT_15_) primers, and ImProm-II™ Reverse Transcriptase (Promega). The relative transcript level of the hp construct was determined via the 2^−ΔΔCt^ method (Livak and Schmittgen 2001) using the following primers (F 5’-GAGACTCTTGCCTACTATGAT-3’ and R 5’-GAGACTTTGCACAATCTTTCTCA-3’) and by normalizing the amount to the strawberry actin (Wang et al. 2016).

### Inoculation test with *B. cinerea* on in vitro detached leaves

A total of 20 leaves, collected from each independent in vitro rooted hp-Bc-DCL1/2 Romina lines, were used to make a preliminary infection test with *B. cinerea* conidia. Five leaves (15 leaflets) for each line were distributed on four Petri dishes containing two sterile paper disks previously imbibed with 2 ml of sterile deionized water. The infection solution, consisting of 0.1 g L^−1^ of PDB, and 10^5^ conidia mL^−1^ of *B. cinerea* B05.10 strain, was dropped (10 μL) on the surface of each strawberry leaflet. 4 days after infection, the relative lesion size (leaflet necrotic area/total leaflet area) was measured on each leaflet through ImageJ software (https://imagej.nih.gov/ij/).

### Inoculation tests with *B. cinerea* on detached strawberry leaves and fruits from hp-Bc-DCL1/2 lines

A total of 12 young leaflets (4 cm diameters) were collected randomly from a minimum of five plants for each of the 3-month-old strawberry independent hp-Bc-DCL1/2 line plus the wild-type control, growing at greenhouse conditions. Each explant was sterilized with 70% ethanol (V/V) solution for 1 min, then washed three times with sterile distilled water. Leaflets were then transversely incised twice to improve the infection efficacy, and inoculated with *B. cinerea* strain B05.10, collecting the conidia previously cultured on Petri dishes filled with MEA. The fungal inoculum was diluted in the infection solution containing 0.1 g L^−1^ Potato Dextrose Broth supplemented with 0.05% Silwet L-77 to a final concentration of 1 × 10^6^ conidia mL^−1^ for spray inoculation test on strawberry leaflets. The relative lesion size, expressed as the ratio of the necrotic surface compared to the control, caused by *B. cinerea* infection, was measured on each sample through the ImageJ software after 4 and 7 days post-inoculation (dpi).

During the second growing season of the hp-Bc-DCL1/2 and wild-type control strawberry lines, in April 2022, a minimum of six mature fruits from five plants for each line were harvested and used for the inoculation test with *B. cinerea*. Fruits were sterilized by dipping them in 10% (V/V) commercial bleach solution (2% sodium hypochlorite) for 30 s, followed by three washes with sterile distilled water. For fruits, the drop inoculation system was exploited, using 10 μL spore suspension diluted to a final concentration of 1 × 10^5^ spores/ml. The relative lesion size on fruits was calculated as described above after 4 dpi; at the same time, 1 cm^2^ of tissue around the inoculum point, and the same amount of tissue from a non-treated area, were collected from each fruit for molecular analyses.

### Data acquisition and statistical analysis

Data on relative lesion size on in vitro and in vivo leaves, and fruits detached from hp-Bc-DCL1/2 lines were analyzed by one-way ANOVA, and the Student–Newman–Keuls test (*p* < 0.05) was used to identify significant differences.

### Assessment of fungal development in hp-Bc-DCL1/2 strawberry fruits

At 4 days after inoculation, an area of 1 cm^2^ around the inoculation site was excised from each of the six fruits for each hp-Bc-DCL1/2 line and the wild-type control, then frozen in liquid nitrogen. DNA isolation was performed using CTAB lysis buffer (0.1 M Tris–HCl (pH 8.0); 1.4 M NaCl; 20 mM EDTA, 2% CTAB, 1% PVP, 0.192 M β-mercaptoethanol). Samples were incubated at 65 °C for 1 h in the presence of RNAse (10 mg ml^−1^). After the addition of chloroform, the samples were centrifuged at 13,000 × g for 10 min at 4 °C. The aqueous phases were recovered and mixed with an equal volume of isopropanol. After incubation on ice for 20 min, the DNA samples were precipitated by centrifugation at 13,000 × g for 30 min at 4 °C. 0.5 μg of DNA was used as a template in a 25 μL reaction using 2X Luna® Universal qPCR Master Mix (Biolabs). Quantitative Real-time PCR (qPCR) reactions were performed on Quant Studio 3 (Applied Biosystems) using the primers reported by Wang et al. (2016). The abundance of *B. cinerea* Internal Transcribed Spacer (ITS) of fungal ribosomal DNA was normalized to the strawberry actin gene.

### *B. cinereaDCL1* and *2* gene transcript quantification in inoculated strawberry fruits

cDNA was synthesized in a total volume of 20 µl from control and hp-Bc-DCL1/2 infected strawberry fruits using 3 μg of total RNA, and following the conditions reported in paragraph 2.4. To evaluate the silencing of *BcDCL-1* and *BcDCL-2*, 1 μL of cDNA was used as a template in a 12.5 μL PCR reaction. The levels of the target transcripts were determined by normalizing the amount to that of BcActin using the primers reported by Wang et al. (2016).

## Results

### Exogenous application of Bc-DCL1/2 dsRNA to control *B. cinerea* infection on strawberry plants grown in the greenhouse

The effectiveness in controlling *B. cinerea* of topically applied naked dsRNAs molecules targeting the *DCL1/2* genes, already observed by Wang et al. (2016) on detached strawberry fruits, was here validated in strawberry plants grown in greenhouse under cultivation condition.

In a preliminary trial, strawberry plants were treated with the same dsRNA molecules at concentrations ranging from 10 up to 50 ng μL^−1^ (Sabbadini et al. 2021b). The highest concentration seemed to be the most effective against the target pathogen, thus we further explored the effect of naked dsRNAs at higher concentrations (50, 75, and 100 ng μL^−1^), compared with the commercial fungicide SWITCH (Syngenta) (positive control). As negative controls, dsRNA molecules targeting part of the sequence encoding for the eGFP (not expressed in the strawberry plants), plus a treatment with only water, were used.

One day after the application of the RNAi-based and control treatments, plants were inoculated with *B. cinerea*, and data on the disease severity on fruits were collected at different time intervals (4, 7, and 14 days).

**Fig. 1:**
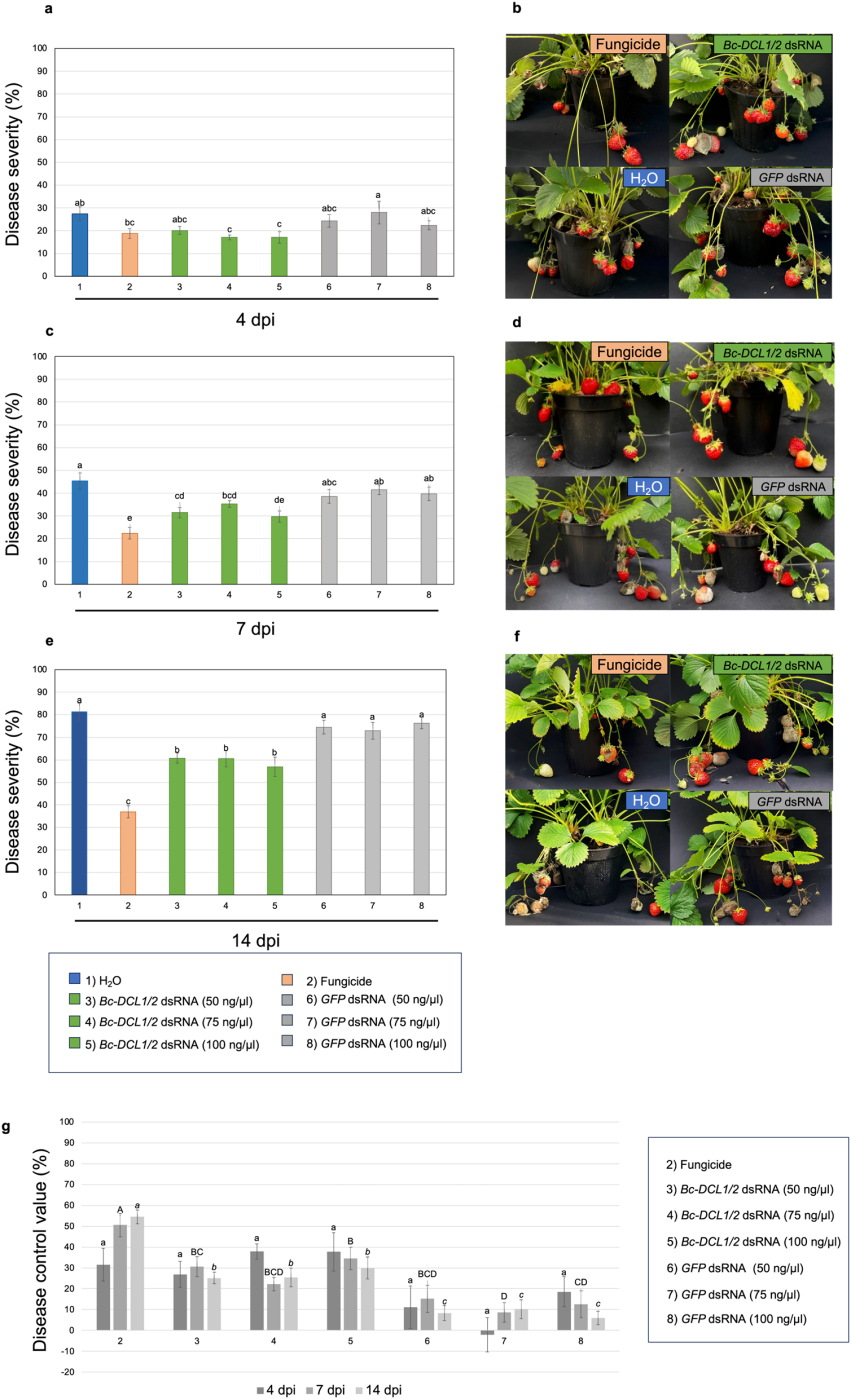
Effect of exogenous application of *Bc-DCL1/2* dsRNA molecules on plants of strawberry cultivated in the greenhouse for the control of *B. cinerea* disease. Disease severity observed after **a** 4, **c** 7, and **e** 14 dpi on plants treated with 50, 75, and 100 ng μL^−1^ of *Bc-DCL1/2* dsRNA or of GFP dsRNA molecules, compared with commercial fungicide and water treatments; representative photos of strawberry plants from each type of treatment after **b** 4, **d** 7 and **f **14 dpi. Disease control value (%) exerted by fungicide and dsRNA-based treatments of gray mold on strawberry fruits observed at 4, 7, and 14 days after *Botrytis* inoculation in the greenhouse. Values followed by small letters (a, b, c) compare data at 4 dpi, by capital letters (A, B, C) at 7 dpi, by italics small letters (a, b, c) at **g** 14 dpi. Means with different letters are significantly different according to the Student Newman-Keuls test (*p* ≤ 0.05) ± SE. Error bars represent the standard errors of three replications

In general, the *Bc-DCL1/2* dsRNA treatments showed a similar efficacy in controlling *B. cinerea* after 4, 7, and 14 dpi, regardless of the dsRNA concentration used, with weaker disease symptoms on dsRNA treated fruits compared to the negative controls (*GFP* dsRNA and water) (Fig. 1). In addition, the percentage of disease severity in the fruits treated with *BcDCL1/2* dsRNA was similar with that observed on strawberry plants treated with the fungicide at 4 dpi, with disease severity values ranging from 17 to 20% and 18%, for the *BcDCL1/2* dsRNA and fungicide, respectively (Fig. 1a).

Disease symptoms increased in all the inoculated plants up to 14 dpi, however on negative control fruits, treated with *dsRNA-GFP* molecules and water, gray mold symptoms and *B. cinerea* conidia sporulation was more accentuated (Fig. 1b, d, f). At 14 dpi, a clear distinction among groups of treatments was observed, both in terms of disease symptoms and disease control value (Fig. 1e, g). In particular, the lowest disease severity value (around 36%) was observed on fruits belonging to plants treated with the commercial fungicide, followed by *Bc-DCL1/2* dsRNA treatments with disease values ranging around 56–60%, and by the negative controls, which showed the highest disease severity values ranging from 72% up to 81%.

The calculated disease control value percentages for the different treatments mirrored disease severity trends. Over time, the commercial fungicide differed as the most effective treatment in controlling the development of gray mold on strawberry fruits (Fig. 1g). Although no significant differences were found at 4 dpi among *BcDCL1/2* dsRNA-based treatments and the fungicide, the latter was more effective at 7 and 14 dpi, with control values around 50% and 54%, respectively, than the *BcDCL1/2* dsRNA-based treatments with a maximum control value around 30% at 14 dpi. The highest concentration of *BcDCL1/2* dsRNAs (100 ng μL^−1^) seemed to provide an efficacy only slightly enhanced overtime, compared to lower concentrations of the same product. In general, the inefficacy of *GFP*-dsRNAs treatments, used as additional negative control, in controlling *B. cinerea* was evident until 14 dpi (Fig. 1g), and significantly higher especially at 14 dpi compared to the other treatments. This response was observed also when using the negative control with only water.

### Development and molecular characterization of hp-Bc-DCL1/2 strawberry lines

**Fig. 2:**
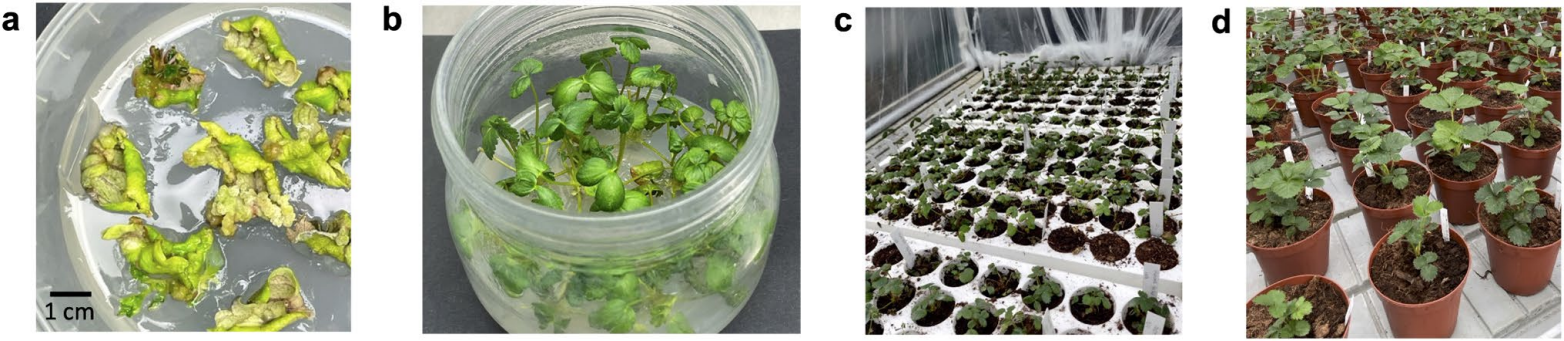
hp-Bc-DCL1/2 strawberry lines production. **a** In vitro regeneration, **b** elongation and rooting of hp-Bc-DCL1/2 strawberry lines. **c**, **d** hp-Bc-DCL1/2 strawberry lines in vivo acclimatization in the greenhouse

Starting from about 100 in vitro expanded leaf explants of strawberry cv Romina, a total of 11 regenerating shoots on selective medium were obtained, then proliferated in vitro (Fig. 2a, b), rooted and subsequently analyzed by PCR analysis to confirm the presence of the hp-Bc-DCL1/2 construct. Results showed the amplification of 340 bp using primers spanning the sequence of 35S promoter in 10 of the 11 regenerated lines (Supplementary Fig. S2). The 10 lines were successively in vivo acclimatized in greenhouse-controlled conditions (Fig. 2c, d).

**Fig. 3:**
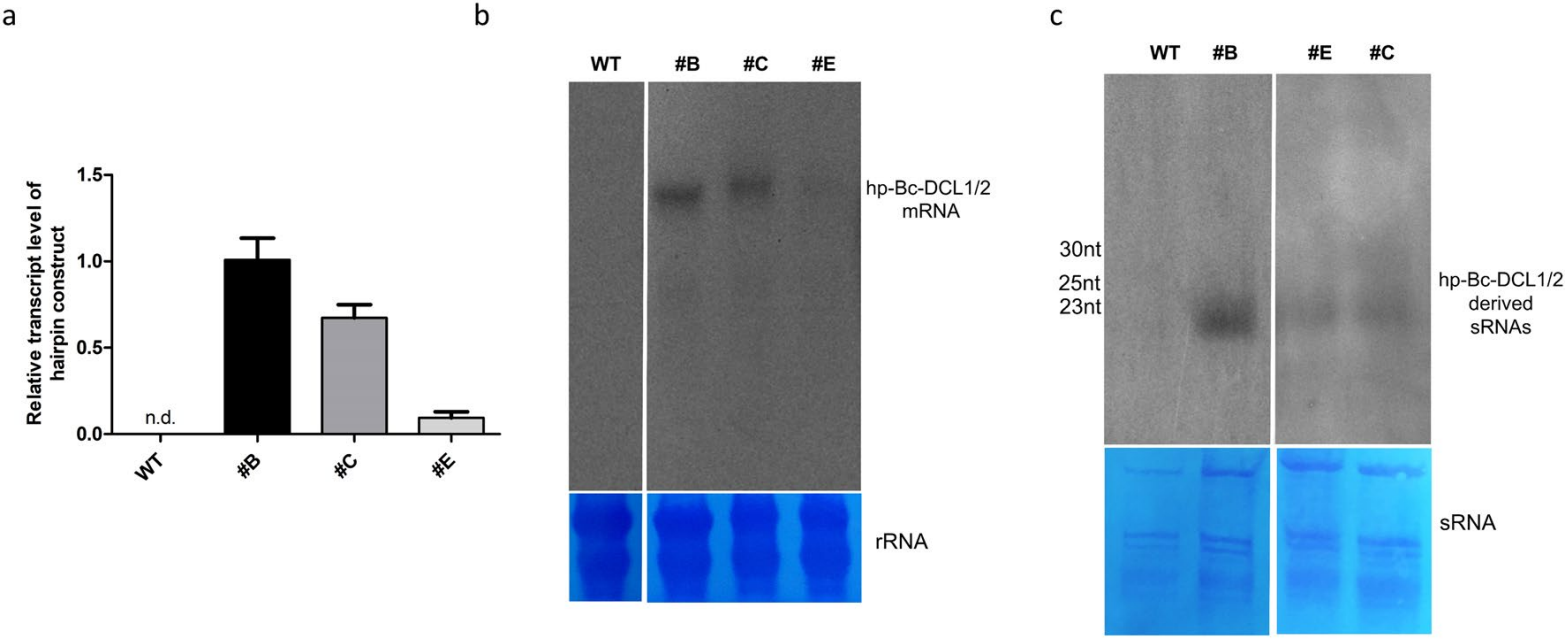
Expression analysis of hp-BcDCL1/2 strawberry lines in the absence of *B. cinerea* infection. **a** qRT-PCR analysis; **b** Northern blot analysis of hp-BcDCL1/2 transcript; and **c** Northern blot analysis of hp-BcDCL1/2-derived siRNAs. The values reported in panel **a** are means ± SE (*n* = 3); n.d. means “not detectable”. Staining with 0.1% toluidine blue of the rRNAs and low molecular weight sRNAs on nylon membranes as a control of homogenous transfer of the loaded RNA samples

Among the 10 hp-Bc-DCL1/2 lines, three lines named #B, #C and #E were selected for their higher plant homogeneity and proliferation and assessed for the expression of the RNAi construct and the production of hp-BcDCL1/2-derived siRNAs before proceeding with the infection experiments. Leaves from 3-month-old plants of the three hp-BcDCL1/2 lines were used for qRT-PCR and Northern blot analyses. All the lines were confirmed for the hp-BcDCL1/2 expression, but at different levels, with the lowest expression observed for line #E (Fig. 3a). The expression of the full-length mRNA of the expected size was ascertained by Northern blot analysis (Fig. 3b). To detect the presence of silencing effector molecules (siRNAs), we analyzed the accumulation of specific siRNAs using a riboprobe designed to target one arm of the hp-Bc-DCL1/2 construct (Fig. 3c). All the lines, in the absence of fungus infection, produce siRNAs around 21–24 nt derived from the processing of the hp-Bc-DCL1/2 transcript.

### Decreased susceptibility to *B. cinerea* of in vitro and in vivo hp-Bc-DCL1/2 strawberry lines

#### Preliminary disease assay on leaflets detached from hp-Bc-DCL1/2 in vitro lines

**Fig. 4:**
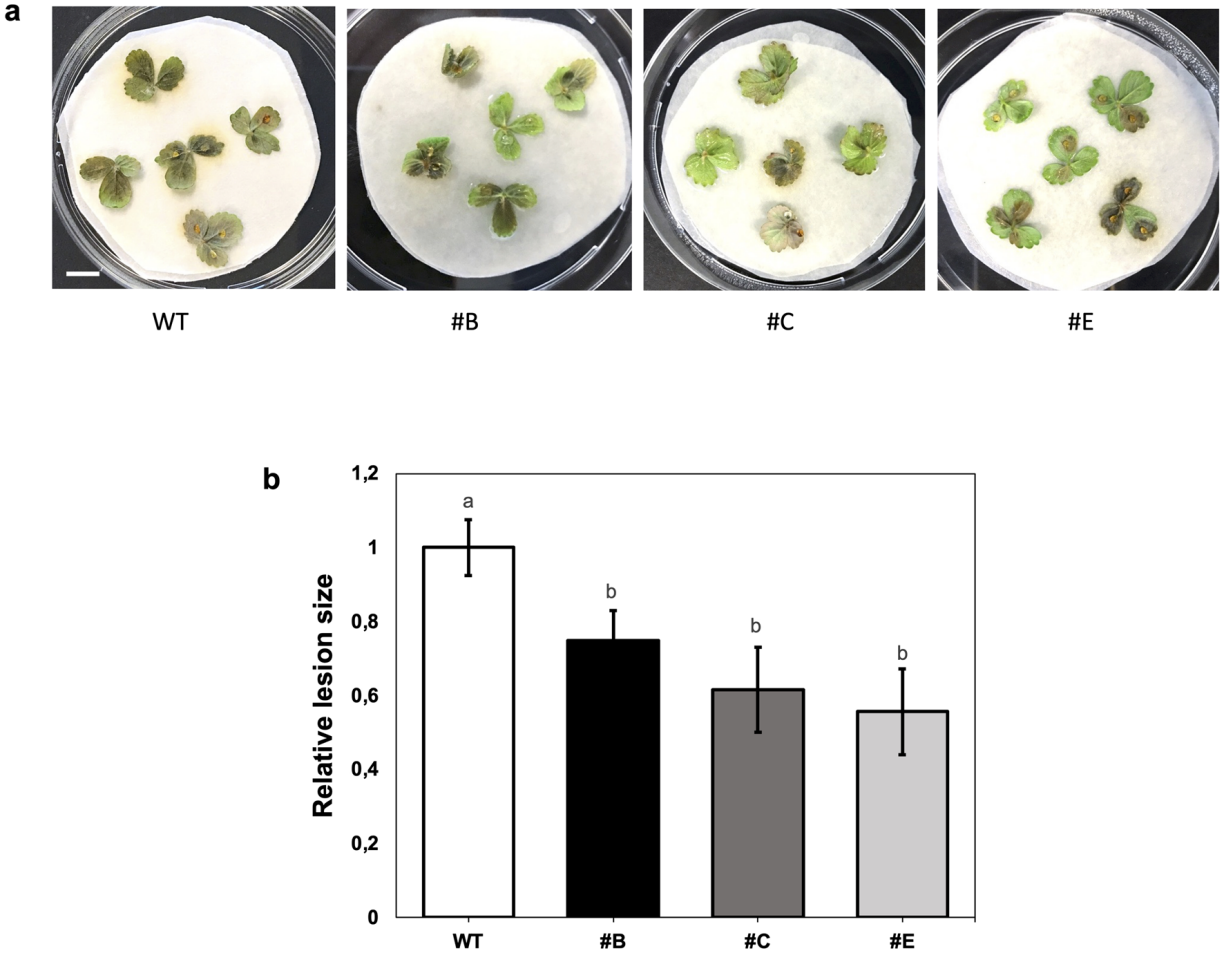
Early screening test of the tolerance of in vitro leaflets from in vitro hp-Bc-DCL1/2 lines. **a** *B. cinerea*-related symptoms on inoculated WT and hp-Bc-DCL1/2 leaflets after 4 dpi. **b** The relative lesion size was measured on strawberry leaflets after 4 dpi. Means with different letters are significantly different according to the Student–Newman–Keuls (*p* ≤ 0.05) ± SE (*n* = 60). Bar = 1 cm

As a preliminary screening, single leaflets of three independent hp-Bc-DCL1/2 in vitro lines, and from wild-type (WT) plant as control, were inoculated with the fungal pathogen. After 4 dpi, leaflets from the three hp-Bc-DCL1/2 lines showed significantly less disease symptoms compared to the WT control, by measuring necrotic lesion size on each explant (Fig. 4a, b).

#### Disease assay on leaves detached from hp-Bc-DCL1/2 lines growing in the greenhouse

**Fig. 5:**
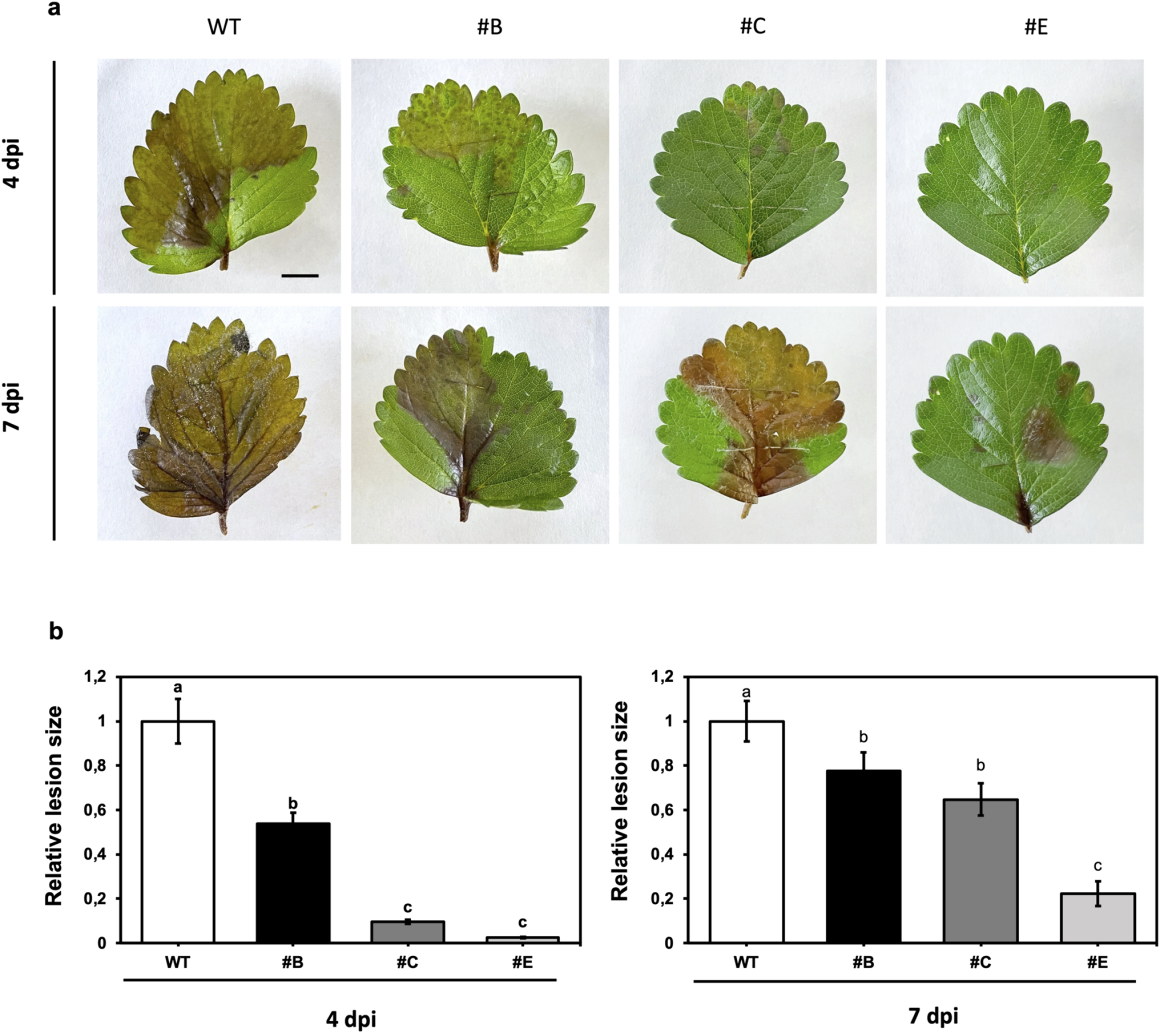
Decreased susceptibility of detached strawberry leaves expressing hp-Bc-DCL1/2 construct after *B. cinerea* inoculation. **a** Necrotic area on inoculated detached leaves of #B, #C, and #E hp-Bc-DCL1/2 lines compared to the WT control at 4 and 7 dpi, and **b** the relative lesion size measured on inoculated detached leaves belonging to #B, #C, and #E hp-Bc-DCL1/2 lines compared to WT control at 4 and 7 dpi. Means with different letters are significantly different according to the Student–Newman–Keuls (*p* ≤ 0.05) ± SE (*n* = 12). Bar = 1 cm

To assess the effect of hp-Bc-DCL1/2 expression on *B. cinerea* virulence, a first infection test was performed on lines #B, #C, and #E using young leaves detached from 3-month-old strawberry plants cultivated in the greenhouse. Lesion area produced by the fungus infection on the hp-Bc-DCL1/2 lines was compared with those on WT control at 4 and 7 dpi (Fig. 5a), and the relative lesion size was measured through Image J software (Fig. 5b). At 4 dpi, all the hp-Bc-DCL1/2 lines showed significantly reduced necrotic area compared to the control, especially in line #C, and #E which showed a reduction in lesion size from approximately 90 up to 97%. In all the hp-Bc-DCL1/2 lines and the WT control, the necrotic area increased on the leaf surface at 7 dpi, but to a significantly lesser extent on the hp-Bc-DCL1/2 lines, which showed a significantly decreased susceptibility to the pathogen. Line #E showed the lowest lesion size, around 77%, compared to the control, in which, on the contrary, conidia sporulation was also observed (Fig. 5a, b).

Lines #B, #C, and #E, which showed different representative levels of tolerance to *B. cinerea* (high, intermediate and low), have been grown until fruit production, to further investigate the efficacy of the siRNAs produced by expression of the hp-Bc-DCL1/2 in limiting the development of *B. cinerea* infection also on fruits.

#### Disease assay on fruits detached from hp-Bc-DCL1/2 lines growing in the greenhouse and downregulation of *Bc-DCL1/2* genes

**Fig. 6:**
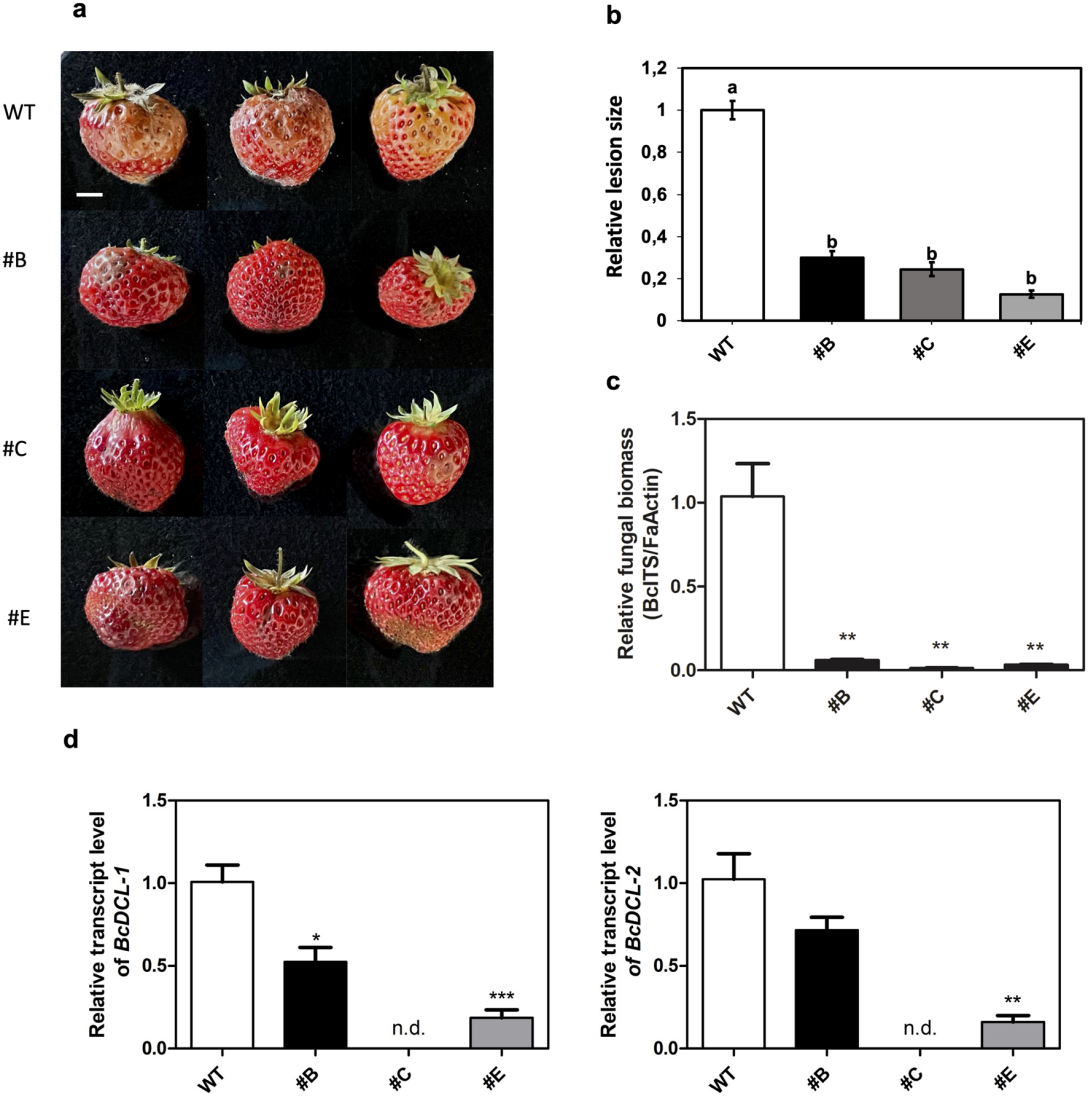
Reduced symptoms caused by *B. cinerea* in hp-Bc-DCL1/2 fruits targeting *DCL1/2* genes. **a** Detached fruits from #B, #C, and #E hp-Bc-DCL1/2 lines compared to WT control, and **b** the relative lesion size measured after 4 dpi; **c** Relative fungal biomass analyzed by qPCR in inoculated fruits belonging to #B, #C, and #E hp-Bc-DCL1/2 lines and the WT control at 4 dpi; **d** *BcDCL-1* (on the left) and *BcDCL-2* (on the right) gene transcript quantification in inoculated fruits belonging to #B, #C, and #E hp-Bc-DCL1/2 lines and the WT control at 4 dpi, measured by qRT-PCR. For panel **b** means with different letters are significantly different according to the Student–Newman–Keuls (*p* ≤ 0.05) ± SE (*n* = 6). For panels **c** and **d**, the values reported are means ± SE (*n* = 3); n.d. means “not detectable”. Student’s t-test was applied to compare differences between hp-Bc-DCL1/2 lines and WT (*p < 0.05; **p < 0.01). Bar = 1 cm

Lines #B, #C, and #E hp-Bc-DCL1/2 growing in the greenhouse reached the fruiting stage, and ripe fruits were harvested and infected in controlled conditions with *B. cinerea*. After 4 days, all the hp-Bc-DCL1/2 fruits developed weaker disease symptoms than WT fruits (Fig. 6a, b) with a reduction in lesion size of approximately 70, 75 and 87% in lines #B, #C, and #E, respectively. In addition, strawberry fruits transformed with a hairpin sequence against a non-intended target were also infected with *B. cinerea* in the same conditions as the other samples (mock control). All fruits showed diffused symptoms caused by the fungal pathogen, similarly to the wild-type control (data not shown). To verify that the reduced lesion size observed in hp-Bc-DCL1/2 fruits was associated to a diminished growth of *B. cinerea*, we assessed the fungal biomass on infected tissues, by determining the abundance of *B. cinerea* Internal Transcribed Spacer (ITS) of fungal ribosomal DNA normalized with strawberry actin (Fig. 6c). Relative fungal biomass was markedly reduced (by approximately 94 to 99%) in the hp-Bc-DCL1/2 fruits demonstrating the significant inhibition of *B. cinerea* growth.

To further confirm that inhibition of fungal development was caused by silencing of the *BcDCL1* and *BcDCL2*, qRT-PCR analysis was performed on total RNA isolated from fruit tissues sampled around the infection sites. To specifically monitor the expression of *BcDCL1/2* and prevent hp-Bc-DCL1/2 expression from influencing the qRT-PCR data, we produced amplicons containing sequences not included in the hp construct (Wang et al. 2016). The expression of *BcDCL1* and *2* genes was not detectable in line #C and was reduced by approximately 50 and 30% in line #B, respectively; in line #E both genes were downregulated by approximately 80% (Fig. 6d). Globally, these data confirm that the silencing of *BcDCL1* and *BcDCL2* genes led to the suppression of fungal virulence and growth.

## Discussion

Small interfering RNAs (siRNAs) are known to regulate several processes in the plant cell, from mRNA degradation and translation inhibition of specific genes, to RNA-directed DNA methylation (Sarkies and Miska 2014). RNAi is a promising disease management strategy against several pathogens and pests, both when dsRNAs are stably expressed in the host plant and, more recently, when exploited as biomolecule applied exogenously to plant tissues. In both of these approaches, essential fungal genes of the target pathogens are commonly selected for silencing (Koeppe et al. 2023). Several researches have already demonstrated in different crops the efficacy of these two strategies against several fungal pathogens, like *B. cinerea*, *Fusarium* spp., *Magnaporthe oryzae* and *Sclerotinia sclerotiorum*, with different levels of tolerance based on the type of pathosystems (Koch et al. 2016; Andrade et al. 2016; Nerva et al. 2020; Wang et al. 2020; Gebremichael et al. 2021; Islam et al. 2021; Jin et al. 2024).

In a previous work, it has been demonstrated that *B. cinerea* delivers small RNAs, produced by BcDCL1 and DCL2 enzymes, into plant cells to silence host immunity genes (Wang et al. 2016). The contribution of these two genes to *B. cinerea* virulence was also supported by infecting several plant species with *Δbcdcl1/2* double knock-out fungal mutants (Wang et al. 2016). In the same work, the authors proved that applying sRNAs or dsRNAs that target *BcDCL1* and *BcDCL2* genes at a concentration of 20 ng μl^–1^, directly on detached strawberry fruits, significantly inhibited gray mold disease by reducing pathogen infection up to 5 dpi (Wang et al. 2016).

The present study aimed to further investigate whether the exogenous application of naked *BcDCL1/2* dsRNA, at concentrations from 50 ng up to 100 ng μL^−1^, could control *B. cinerea* infection on whole strawberry cv Romina plants grown in greenhouse under cultivation condition up to 14 dpi. The results showed a significant level of protection against *B. cinerea* infection of fruits by plants treated with *BcDCL1/2* dsRNA up to 14 dpi (control value around 30%), compared with negative controls, but lower than the commercial fungicide (control value around 54%)(Fig. 1g). In addition, no significant differences were detected with increasing dsRNA concentrations tested. In another study, the dose–response of exogenous dsRNAs targeting various genes in *S. sclerotiorum* grown in liquid culture was evaluated. Results showed that increasing concentrations of dsRNA did not result in increased transcript reduction of some of the targets (McLoughlin et al. 2018). In another greenhouse trial performed on potato plants aimed at validating a new RNAi-based product (Ledprona®) against the Colorado potato beetle, increasing rates of this formulation showed no differences in the insect survival percentage (Rodrigues et al. 2021).

As already hypothesized by McLoughlin et al. (2018), the RNAi silencing machinery could become saturated at specific dsRNA concentrations, preventing the processing of excess molecules and the resulting apparent lack of increasing efficacy at increasing dsRNA concentrations.

The experiments performed in our study are fundamental to further validate the efficacy of dsRNA sequences previously tested only on detached plant tissues at laboratory level (Wang et al. 2016), also on cultivated strawberry plants now grown in greenhouse and to study the effect of different concentrations to contain the fungal infection in specific pathosystems. By comparing the effect of the commercial fungicide (better performing at longer time intervals) and that of naked dsRNA, it can be suggested that the instability of these molecules in the long period represents the main limitation of this RNAi-based strategy as a possible alternative bio-fungicide. This response confirms the need to produce a new formulation, such as those based on nanoparticles/nanomaterials exploited as carriers of target dsRNA molecules, which can increase the stability and efficiency of the molecules while improving the ability of dsRNA to penetrate the target cell (Islam et al. 2021; Ray et al. 2022; Niño-Sánchez et al. 2022; Qiao et al. 2023; Scarpin et al. 2023). In the study by Islam et al. (2021), the topical application at a low concentration (1 ng μL^−1^) of naked or formulated dsRNA (encapsulated in *E. coli*-derived anucleated minicells, ME-dsRNA) targeting the same *Bc-DCL1* and *2* genes, was performed on single fruits of plants grown in greenhouse one hour before *B. cinerea* mycelium plug inoculation. Minimal fungal growth was observed on the fruits treated with naked dsRNA molecules, but better results were obtained when the ME-dsRNA were used, which completely inhibited disease progression at 5 days post-inoculation. These formulated dsRNA molecules were also able to extend the protection window against *B. cinerea* by 12 days compared to naked dsRNA, when the pathogen was inoculated 7 days post-treatment, confirming the potential of nanocarriers to improve dsRNA stability and effectiveness in greenhouse or field condition.

Certainly, a valid alternative to any sprayable product is the availability of new varieties with stable resistance to target pathogens and pest. Therefore, we tested the effectiveness of stable expression of *BcDCL 1/2* dsRNA in plants to reduce the pathogen infection in cultivated strawberry. New hp-Bc-DCL1/2 strawberry lines were produced and molecularly characterized in the absence of *B. cinerea* infection. Leaves harvested from three hp-Bc-DCL1/2 independent lines, showed stable expression of the hp-Bc-DCL1/2 gene construct. The production of hp-BcDCL1/2-derived siRNAs was also verified in these lines by Northern blot analysis, confirming the activation of the RNAi machinery even in the absence of fungal infection.

A method for early evaluation of the efficacy of stable expression of the hp-Bc-DCL1/2 construct in controlling the *B. cinerea* pathogenicity was developed by infecting detached leaves from plants still in vitro and maintaining them in Petri dishes under high humidity conditions. This disease assay allowed us to immediately distinguish the hp-Bc-DCL1/2 lines from the WT control due to a significant reduction in *B. cinerea* growth at early stages of infection (4 days of leaves incubation). The same result was also observed from the inoculation of *B. cinerea* on leaves detached from hp-Bc-DCL1/2 expressing lines grown in the greenhouse. In fact, all hp-Bc-DCL1/2 lines showed a significantly reduced susceptibility to the pathogen when compared to the WT control, up to 7 dpi.

*B. cinerea* is extremely aggressive on strawberry fruits. To assess RNAi-strawberry fruits susceptibility, the hp-Bc-DCL1/2 expressing lines were grown in the greenhouse up to fruiting stage. Ripe fruits were harvested and inoculated with fungal conidia. The expression of hp-Bc-DCL1/2 gene construct was effective also in reducing damages of *B. cinerea* infection on fruits compared to the WT control up to 4 dpi, with no significant differences in relative lesion size among the different hp-Bc-DCL1/2 lines. Quantification of *B. cinerea* genomic DNA by qPCR demonstrated a significant reduction in mycelial growth on the inoculated hp-Bc-DCL1/2 fruits compared to the WT control, thus demonstrating the effect of harpin gene expression in the inhibition of fungal pathogenicity. Transcript quantification of *B. cinerea DCL1* and *2* performed in inoculated strawberry fruits demonstrated the downregulation of the target genes, confirming that the suppression of fungal virulence and growth was due to the silencing effect of dsRNAs. Among the lines, line #E showed lesions similar to those of other hp-Bc-DCL1/2 lines in the fruits, but a greater capacity of controlling *B. cinerea* infection in leaves, even if characterized by the lowest hp-Bc-DCL1/2 transcript level and by a weak siRNA accumulation in the absence of infection. As shown by other authors, the production of siRNA can vary after pathogen infection because of the presence of the dsRNA-targets and also for the amplification of the silencing signal via transitivity (Koch et al. 2013). Similar findings were reported in other studies performed on different pathosystems (plant-fungi), in which a direct correlation between transgene expression or hp-construct-derived siRNAs accumulation, and resistance to the target pathogen has not been observed (Nowara et al. 2010; Ghag et al. 2014). However, further investigation could be useful to discover possible correlations between siRNA abundance and induced resistance to target pathogen in RNAi-based plant systems.

Previous studies aimed at the production of new strawberry cultivars resistant to fungal diseases, have identified and validated resistance genes (R genes) in this crop (Pincot et al. 2018; Barbey et al. 2019; Ma et al. 2023). However, due to the intrinsic soft texture of strawberry fruits, R genes hardly confer acceptable resistance to gray mold on fruits, but only on vegetative parts. Indeed, the resistance based on R gene is laborious to obtain and can only be exploited via genetic introgression through conventional breeding protocols or new genomic techniques. Moreover, R gene resistance is generally neither stable nor durable because of the strong selective pressure exerted on pathogen populations and the consequent emergence of new strains insensitive to the R gene action (Stam and McDonald 2018). The data presented in our study demonstrate, for the first time in the cultivated strawberry, the efficacy of RNAi-based approaches to strongly control *B. cinerea* growth on fruits that are normally damaged by this pathogen in few days.

Furthermore, this represents one of the few studies which exploited the stable expression of dsRNA against fungal diseases in a perennial species, showing a strong reduction in susceptibility on plant tissues.

In some of the hp-BcDCL1/2-strawberry lines, fruits were protected from gray mold by more than 80% (reduction in lesion size) up to 4 dpi. This result seems more promising than that observed with the application of the naked dsRNA sequences at the same time interval, where a disease severity control value ranging from 27 to 38% was observed, and especially than the protection level observed with chemical pesticide application (around 32% of disease severity control value). This suggests that the expression of target dsRNA in plants could be the best solution to protect plants from *Botrytis*, offering the possibility to drastically reduce the need for specific treatments for controlling this fungus. However, further studies should be performed in which the two RNAi-based strategies are compared under the same experimental conditions. In analogy to our research, Dou et al. (2020) demonstrated the high efficacy of this approach in providing resistance in a perennial species, specifically to *Fusarium* wilt in banana, by targeting vital genes of the pathogen through stable expression of a hairpin construct in plant. In that study, the production of hairpin-derived siRNAs ranging from 21 to 24 bp was also verified, with the small 21 bp RNAs being the most abundant in the RNAi-banana plants. As for many other studies exploiting a similar strategy, the efficacy in controlling pathogen growth can also be linked to the use of the 35S promoter, which drives the constitutive expression of the hairpin construct in all plant tissues. It is known that this promoter is transcribed by RNA Pol II, and that hairpin sequences transcribed by this enzyme are mainly processed by DCL4 in the plant, with the consequent production of 21-nt siRNAs, known to mediate post-transcriptional gene silencing within the plant, and most probably during plant–pathogen interaction (Elbashir et al. 2001; Zand Karimi and Innes 2022). However, further investigations will be necessary to confirm which of the produced hp-derived siRNAs primarily act as silencing molecules in our specific pathosystem, for example by analyzing siRNA profiles through sRNA-seq.

## Conclusions

Overall, these results support previously reported evidence (Koch et al. 2016; Wang et al. 2016; Werner et al. 2020; Haile et al. 2021; Islam et al. 2021) that the knockdown of key genes of the RNAi fungal machinery, such as *DCL* genes, is a good strategy to negatively affect the pathogenesis of devastating fungi such as *B. cinerea*.

We have demonstrated that, as an alternative to traditional synthetic and biological pesticides, it is possible to control *Botrytis* infection in cultivated strawberries using RNAi technology, both through the production of new sprayable products and, for the first time, by producing resistant plants.

**Fig. 7:**
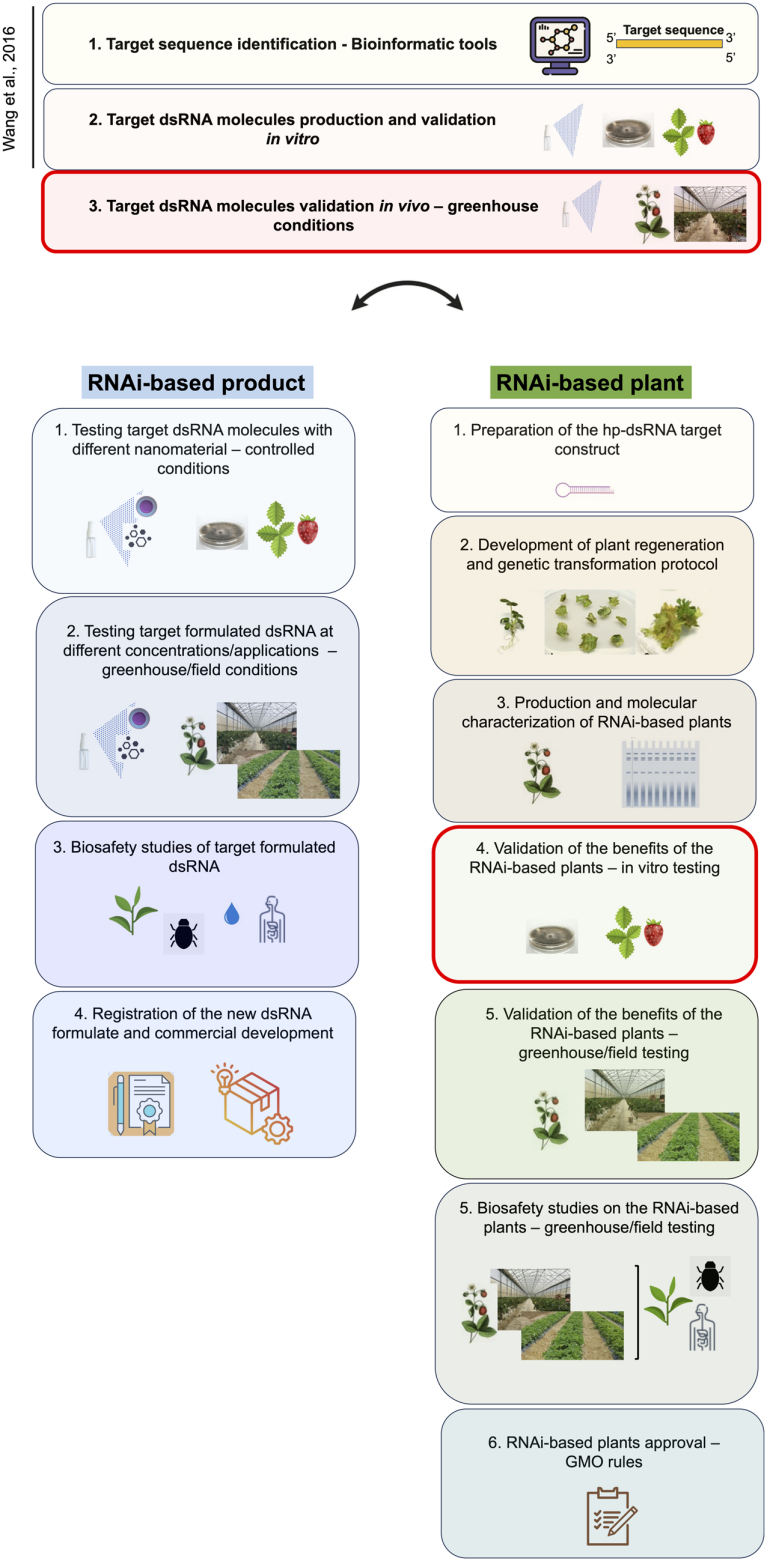
Experimental and regulatory steps required for the development of RNAi-based products and plants. The first three steps are common and necessary to both RNAi-based strategies. In the specific pathosystem described in our study (Strawberry—*B. cinerea*), steps 1 and 2 have already been performed by Wang et al. (2016). Red squares underline the steps reached in our work for both pathways

The results obtained in this study are at an already advanced Technology Readiness Level (TRL) in the long process required prior to the registration/approval of an RNAi-based product/plant (Fig. 7). Indeed, the effectiveness of topically applied naked *BcDCL 1/2* dsRNAs to control *B. cinerea* was amply demonstrated at both molecular and applicative level by Wang et al. 2016 on detached strawberry fruits at laboratory level (TRL 2–3). The trial performed in this study was aimed to test these molecules when applied on whole plants grown in a more relevant environment, to preliminarily investigate its effectiveness when used as bio-fungicide (TRL 4–5). The results obtained confirmed the capacity of these molecules in controlling the infection of *B. cinerea* on plants of a strawberry commercial cultivar growing in a relevant environment, such as the greenhouse. The results of this experimentation will contribute to better defining the strategy to produce a new dsRNA-based formulation.

This preliminary validation of the sequence is critical for defining the use of the same RNAi sequences for producing new plants with decreased susceptibility to the target disease, through the stable expression of the selected dsRNA-targeting molecules. In fact, our data also indicate that the *in planta* stable expression of a hairpin construct targeting the same *Bc*-*DCL* genes, can strongly reduce susceptibility to *B. cinerea* infection in a cultivated strawberry cultivar. In addition, these hp-Bc-DCL1/2 lines can provide a new important experimental model for studying the *in planta* mobility of the expressed dsRNA sequences, most likely via EVs, considered as the main siRNA-stabilizing factor during plant-pathogen interaction (Koch and Wassenegger 2021). This new knowledge can contribute to the progress in the development of new defense strategies against *Botrytis* in strawberries but also applicable to other species devastated by this fungus.

The results obtained by comparing the two different RNAi technologies allowed us to quantify their different potential, indicating the *in planta* expression technology as the most encouraging one, offering the real possibility of significantly reduce the application of the more dangerous pesticides. The promising results observed from the exogenous application of dsRNA molecules demonstrate how this strategy is also extremely valid for contributing to the reduction/replacement of chemical pesticides with safer and more sustainable formulations such as those based on dsRNA. The benefits of the new RNAi-based plants or products feature also high biosafety due to the sequence specificity of dsRNA molecules, reducing the risk of off-target or unintended effects in the host and in non-target organisms. Moreover, in cells, the dsRNA molecules subjected to RNAi will be processed in various different specific sRNAs directed to silence one or more target genes, and this mechanism of action is not easily overcome by the onset of new pathogen strains.

Together the two RNAi-based strategies described in this study can strongly contribute to develop more sustainable agronomical solutions and integrated pest management.

However, the next challenge is to achieve the authorization and acceptance of new RNAi-based products and plants. In the case of new RNAi-based formulations, some products are at an advanced stage of authorization (e.g., Calantha™ and Ledprona™ GreenLight Biosciences Inc.), and this process seems easier to manage as it corresponds to the legislation for the authorization of new products classified as chemical or biological pesticides. RNAi-based plants will have to face a more difficult path as, being produced with the recombinant DNA technique, they will have to be evaluated and approved according to the rules for GMO regulation (Arpaia et al. 2020).

### Supplementary Information

Below is the link to the electronic supplementary material.

Supplementary file1 (DOCX 475 KB)

## Data Availability

The original contributions presented in the study are included in the article/supplementary material. Further inquiries can be directed to the corresponding author.
