# Correction to: RNA interference-based strategies to control *Botrytis cinerea* infection in cultivated strawberry

**DOI:** 10.1007/s00299-024-03296-7

**Published:** 2024-09-02

**Authors:** Luca Capriotti, Barbara Molesini, Tiziana Pandolfini, Hailing Jin, Elena Baraldi, Michela Cecchin, Bruno Mezzetti, Silvia Sabbadini

**Affiliations:** 1 Department of Agricultural, Food and Environmental Sciences, Università Politecnica delle Marche, Ancona, Italy; 2 Department of Biotechnology, University of Verona, Strada Le Grazie, 15, 37134 Verona, Italy; 3 Department of Microbiology and Plant Pathology, Center for Plant Cell Biology, Institute for Integrative Genome Biology, University of California, Riverside, CA USA; 4 Department of Agricultural and Food Science, DISTAL, Alma Mater Studiorum - University of Bologna, Bologna, Italy

**Correction to: Plant Cell Reports (2024) 43:201** 10.1007/s00299-024-03288-7

All the authors first and family name were incorrectly swapped in the original publication. The complete correct names are given below.

Luca Capriotti, Barbara Molesini, Tiziana Pandolfini, Hailing Jin, Elena Baraldi, Michela Cecchin, Bruno Mezzetti, Silvia Sabbadini.

The original article has been corrected.

