# Supplementary material for: RNA interference-based strategies to control Botrytis cinerea infection in cultivated strawberry
Source: Plant Cell Rep. 2024 Jul 24;43(8):201. doi: 10.1007/s00299-024-03288-7 (PMC11269516; doi:10.1007/s00299-024-03288-7)
Supplement: Supplementary file 1 — Supplementary file1 (DOCX 475 KB) [file 299_2024_3288_MOESM1_ESM.docx]

**Plant Cell Reports**

**RNA interference-based strategies to control *Botrytis cinerea* infection in cultivated strawberry**

Capriotti Luca, Molesini Barbara, Pandolfini Tiziana, Jin Hailing, Baraldi Elena, Cecchin Michela, Mezzetti Bruno^*^, Sabbadini Silvia^*^

*Corresponding authors

E-mail: Silvia Sabbadini (s.sabbadini@staff.univpm.it); Bruno Mezzetti (b.mezzetti@staff.univpm.it)

Department of Agricultural, Food and Environmental Sciences, Marche Polytechnic University,

Ancona, Italy


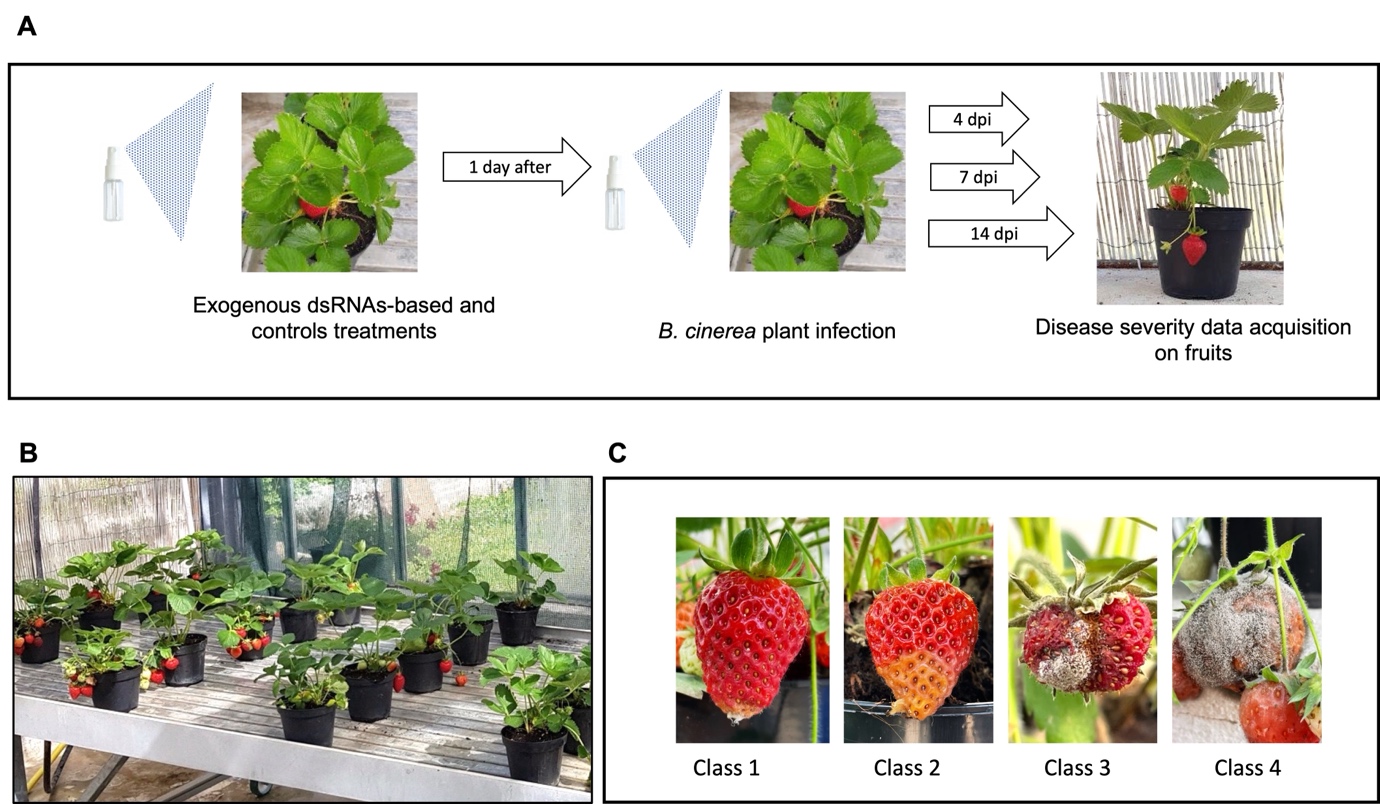


**Fig. S1.** Exogenous application of naked *Bc-DCL1* and *2* dsRNAs in the cultivated strawberry (*Fragaria* x *ananassa*) grown in the greenhouse.

**A** Schematic diagram of the disease assay performed; **B** Image of strawberry plants used for the trial; **C** Class of diseases used to measure the disease severity index after each treatment.


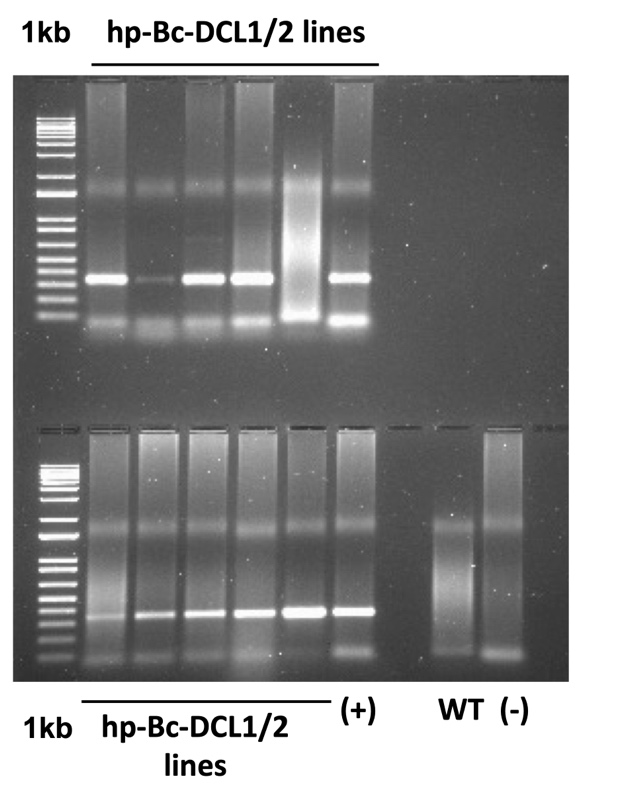


**Fig. S2.** PCR analyses of hp-Bc-DCL1/2 lines.

Amplification of 35S promoter (340 bp) gene fragment from the genomic DNA of 11 hp-Bc-DCL1/2 and one wild-type (WT) lines obtained from different starting leaves explants, and DNA of pHellsgate–*Bc*-*DCL1/2* plasmid “(+)”. The lane labelled “(−)” shows the PCR result using water as negative control. DNA marker (1Kb Plus DNA Ladder, Invitrogen, Carlsbad, CA, USA).
